# Exploratory application of DMD for particle deposition and fluid field in the respiratory tract^☆^

**DOI:** 10.1016/j.jaerosci.2025.106718

**Published:** 2025-11-21

**Authors:** Martin S. Graffigna, Ignacio R. Bartol, Mauricio E. Tano, Shaheen Azim Dewji

**Affiliations:** a Nuclear and Radiological Engineering and Medical Physics Program, George W. Woodruff School of Mechanical Engineering, Georgia Institute of Technology, 770 State St NW, Atlanta, 30332-0405, GA, United States of America; b Collaborative Computing Center (C3), Idaho National Laboratory, 955 MK Simpson Boulevard, Idaho Falls, 83415, ID, United States of America

**Keywords:** DMD, Dynamic Mode Decomposition, Respiratory tract, Fluid dynamics, CFD, Deposition, Reduced order model

## Abstract

Simulating particle deposition in the respiratory tract requires high computational effort due to the intricate airway geometry and complex airflow–particle interactions. To address this challenge, this study introduces the first demonstration of Dynamic Mode Decomposition (DMD) as a reduced-order model to infer the trajectories of inhaled particles during a breathing cycle and to evaluate the applicability of DMD as a fluid field interpolator. The periodic nature of respiration and the predominance of sinusoidal boundary conditions make it well-suited for DMD analysis. Three high-fidelity computational fluid dynamics (CFD) simulations were performed under three different inlet volume airflow conditions for the same realistic adult male anthropomorphic phantom respiratory tract model. Reduced-rank DMD reconstructions were compared to the CFD ground truth, yielding a Mean Relative Error (MRE) of 12% in the velocity field. Additionally, a fourth simulation was conducted at an intermediate point to evaluate the interpolation capability of the parametric DMD framework in complex systems. This interpolation resulted in an MRE of 20%, with the reconstructed flow field capturing dominant fluid modes and overall dynamics, though localized discrepancies reached relative errors up to 70%.

While DMD effectively reconstructed fluid fields, preserving mean flow regimes, some deviations were observed in Lagrangian particle tracking, specifically in spatial deposition resolution. However, the method approximated overall particle distribution with an 85% correlation to ground truth and was effective in representing regional deposition patterns across the tracheobronchial tree. These findings support the utility of DMD a computationally efficient approach for fluid field reconstruction and particle transport analysis in respiratory flow simulations.

## 1. Introduction

Inhalation of aerosolized particles occurs across a range of environments, with elevated exposure risk in occupational settings involving dust, fumes, or hazardous aerosols. When inhaled material contains harmful substances, such as radioactive aerosols or toxic compounds, quantifying deposition patterns in the respiratory tract is necessary for evaluating internal exposure and guiding dosimetric analysis (Bartol et al., 2024). Obtaining high-resolution particle deposition profiles (PDPs) is also relevant in therapeutic applications involving inhaled drug delivery, as these profiles provide spatial information on impacted airway regions and, in the case of radioactive aerosols, inform dose estimation to radiosensitive tissues. Characterizing subject-specific particle distribution within the human airways can improve exposure assessment models, evaluate deposition variability across anatomical geometries, and support individualized simulation frameworks.

While computational fluid-particle dynamics (CFPD) has emerged in recent decades as a tool to assess subject-specific PDPs, as demonstrated by Katz et al. (1999), Kleinstreuer and Zhang (2010), Kleinstreuer et al. (2008), CFPD-based approaches remain a computationally intensive solution. Reduced-order models (ROMs) are an alternative tool for accelerating CFPD simulations by identifying and retaining only the dominant coherent structures in the flow. The concept behind ROMs is to reduce the number of degrees of freedom in the governing equations; this enables a rapid reconstruction of the flow field with minimal loss of fidelity. Multiple data-driven techniques operate by decomposing high-dimensional CFD data into a set of spatial modes that evolve with a set of fixed temporal characteristics. In the study by Habibi et al. (2020), a Dynamic Mode Decomposition (DMD)-based ROM was applied to simulations of pulsatile blood flow in a reduced geometry. By retaining 53 DMD modes, the reconstructed solution achieved approximately 99.5% agreement with the corresponding full-order computational fluid dynamics (CFD) results. This high level of agreement illustrates the capacity of DMD to capture the dominant temporal and spatial features in systems characterized by periodic boundary conditions.

In contrast, the study by Holemans et al. (2022) utilized Spectral Proper Orthogonal Decomposition (SPOD) to develop a ROM for large CFD data sets obtained from simulations of an annular swirling jet. By extracting data from two perpendicular 2D planes instead of processing the full 3D data set, the study achieved reduced computational time, achieving a 68-fold acceleration and reduced memory usage. However, this approach incurred a reconstruction error of ~10% for the relatively simple geometry, modeled with high resolution (> 10 million elements) and with a consistent revolving frequency of 22 Hz. Despite the higher error, the method effectively captured the large-scale coherent structures that govern the main flow dynamics. The ability to isolate and preserve these dominant features makes ROMs well-suited to respiratory flow modeling, where computational cost and geometric fidelity are both important for high-throughput or patient-specific simulation efforts.

In a related computational study, Jing et al. (2024) employed Large Eddy Simulation (LES) to analyze inspiratory airflow dynamics in a CT-derived human respiratory tract model under tidal and quasi-steady flow conditions, validated through grid independence tests. Using DMD on LES-derived vorticity fields, Jing et al. extracted coherent flow structures characterized by single frequencies and explicit decay/growth rates, which enabled an accurate identification of transient mechanisms. As a comparison, Proper Orthogonal Decomposition (POD) was applied, revealing its limitations in isolating frequency-specific dynamics due to energy-ranked modes that combine multiple spectral components. The study further demonstrated that DMD outperformed POD as the choice of ROM in respiratory applications, as its ability to resolve temporally evolving modes, such as turbulence in the pharynx-larynx region, was not captured when using POD. This advantage positions DMD as a more effective tool for dynamic analysis, including particle transport studies, where transient flow interactions dictate deposition outcomes.

To distinguish among the three decomposition methods, the work of Schmidt and Colonius (2020) provides a comparative analysis. POD extracts orthogonal spatial modes by performing an eigenvalue decomposition of the entire time-resolved dataset. These modes are ranked by energy and represent the most spatially coherent structures present throughout the simulation. Because POD does not retain temporal evolution, it produces a purely spatial decomposition that captures dominant features without time-dependent behavior.

In contrast, SPOD first applies a Fourier transform to the time-resolved data to isolate individual oscillation frequencies, followed by an eigenvalue decomposition at each frequency to obtain spatial modes. This frequency-domain approach enables SPOD to resolve structures that are coherent in both space and time, preserving the highest-energy modes at each frequency and linking them directly to the system’s temporal dynamics via the Fourier domain.

Finally, DMD seeks a linear operator that advances the system in time, using singular value decomposition (SVD) as part of the procedure. DMD identifies modes that are not constrained to be orthogonal but are optimized to reconstruct the system’s evolution, often capturing growth or decay rates and oscillatory behavior.

While POD and SPOD emphasize spatial compression using orthogonal basis functions, DMD prioritizes reconstruction of time-dependent system behavior (Towne et al., 2018). DMD modes may overlap spatially, but they are selected to best represent the system’s dynamic trajectory.

The present study investigates the reconstruction capacity of the DMD framework and evaluates its use for interpolating flow fields in highly complex and non-linear systems. Such interpolated fields can then be utilized under various particle deposition calculation schemes, such as the direct solution of uncoupled particle transport equations or more sophisticated data-driven methods.

DMD is a powerful data-driven method that extracts coherent structures and dynamics from high-dimensional systems (Kutz & Brunton, 2016). It is based on the Koopman operator theory, which describes the evolution of observables in a dynamical system. The Koopman operator, Eq. (1), 𝒦, acts on a space of observables g(x) such that:

(1) $$𝒦g\left(x_{k}\right)=g\left(x_{k+1}\right),$$

where $x_{k}$ represents the state of the system at time k. DMD approximates the Koopman operator by decomposing the data into modes that evolve exponentially in time, allowing for the reconstruction and prediction of the system’s dynamics. Mathematically, given a sequence of snapshots of the system state $X=\left[x_{1},x_{2},\ldots ,x_{m}\right]$, DMD, Eq. (2), seeks a linear operator A that best approximates the dynamics:

(2) $$x_{k+1}\approx Ax_{k}.$$

The DMD modes and eigenvalues are then obtained from the eigen-decomposition of A, providing a low-dimensional representation of the system’s behavior.

This study is structured into three phases. The first phase involved the application of DMD to reconstruct particle positions in the respiratory tract by directly applying DMD to the dynamics of particle positions. The second phase examined the reconstruction power of DMD for the fluid field in the respiratory tract and compared it with high-fidelity simulations. The third phase presents the results of interpolating the Koopman operator to reconstruct a fluid field for which the model was not explicitly trained. The interpolation scheme builds upon the work of Huhn et al. (2023), who demonstrated that interpolating the reduced Koopman operator relaxes smoothness constraints and improves fidelity relative to direct snapshot stacking. This three-phase structure provides a basis for evaluating the performance and limitations of DMD and Koopman-based surrogates relative to high-fidelity models.

## 2. Methodology

To reconstruct particle positions and fluid fields, three high-fidelity CFPD simulations were first performed on the same adult male human respiratory geometry, which was reconstructed from CT scans and implemented with two-way coupling between particles and fluid, meaning particles have mass and volume and that particles have feedback on the Navier–Stokes equations. A two-way coupled Lagrangian tracing was performed in StarCCM+ (Software, 2021), using a Lagrangian Multi-phase (LMP) solver.

### 2.1. CFD simulation details

**Fig. 1.**
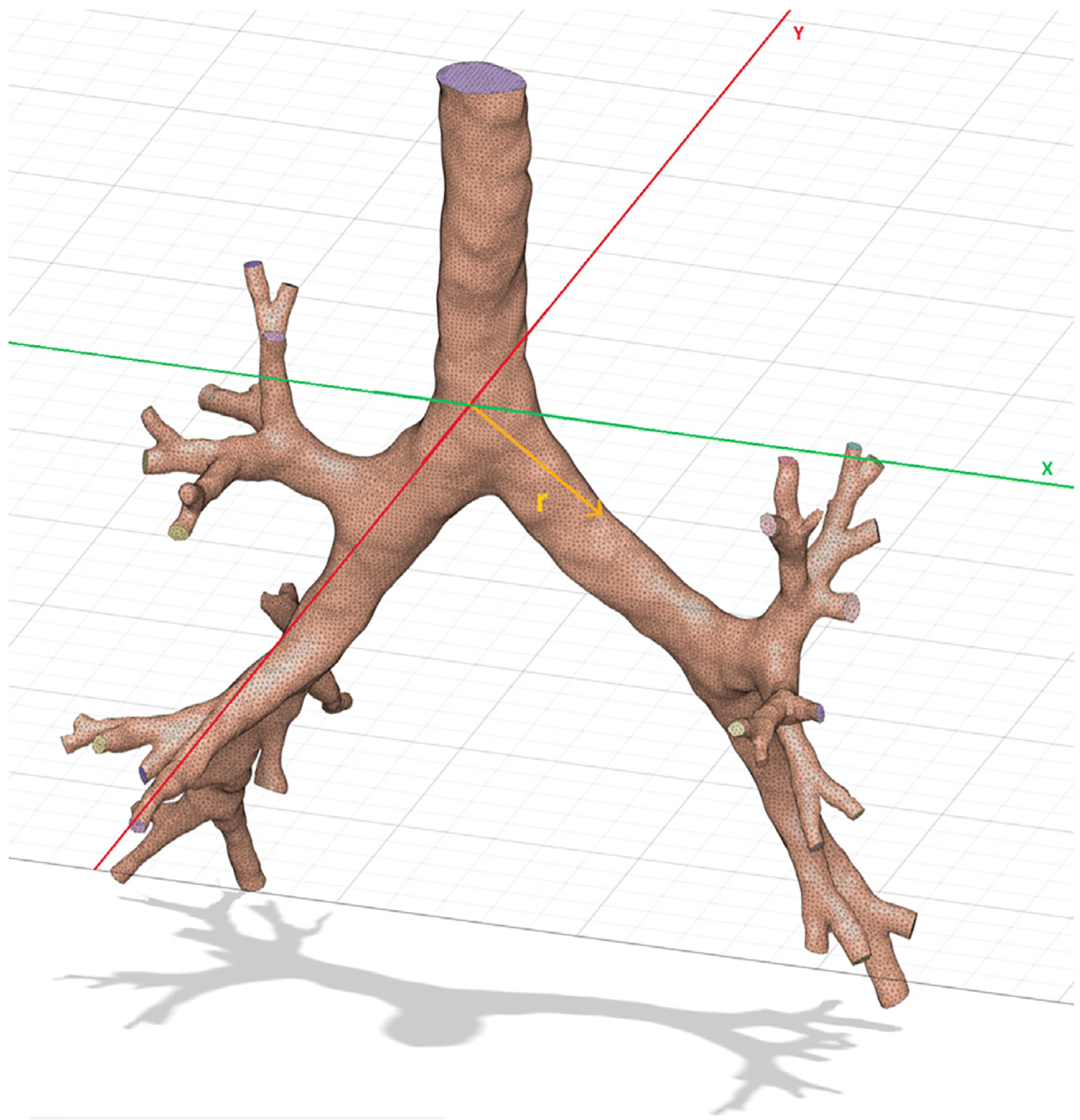
Reconstructed 3D geometry of a human airway, obtained by segmenting the air cavity in each CT scan slice and assembling them in sequential order. The resulting surface was smoothed to make it suitable for CFD simulations. The origin is situated at the ridge separating the right and left main Bronchi, also known as Carina. A distance vector r in spherical coordinates is taken from this point as a measure of distance, shown in orange.

The airway model used represents the lower respiratory tract up to the 5th generation of bronchi. This geometry was reconstructed by segmenting the air cavities of a CT scan deck and then arranged sequentially using a pre-trained neural network from Wang et al. (2022) and post-processed for a CFPD simulation using the methodology adopted from Bartol et al. (2024). A schematic of the resulting geometry can be seen in Fig. 1:

The particle distribution used corresponded to a polydisperse log-normal distribution with mean (μ) and standard deviation (σ) equal to μ=0.42μm and σ=3.5, and a density of $\rho =4390\;\text{kg}/{\text{m}}^{3}$. This particle distribution relates to a form of aerosolized iodine-131, a radioisotope formed as a byproduct in nuclear fission relevant to nuclear accidents and fallout scenarios, which was previously used by Talaat et al. (2019) for CFPD simulations.

The simulation was performed using the licensed finite volume solver StarCCM+ (Software, 2021). The boundary conditions for the velocity $\vec{u}=(u,v,w)$ were chosen to replicate a representative respiratory cycle of a human subject under heavy-exercise breathing conditions, completing a 2.4-second breathing cycle. The inlet flow was computed as in , where t is the time of the simulation, and T is the breathing period, which in this case will be T=2.4s. Lastly, ${\dot{v}}_{\text{max}}$ is the peak air flow in a breathing cycle $\dot{v}(t)={\dot{v}}_{\text{max}}\;\text{sin}\;\left(\frac{2\pi t}{T}\right)$.

The mesh consisted of polyhedral elements, with four boundary layers and a base element size of 1.5 mm. Furthermore, refinement of the cells near the wall region was performed, resulting in an element size of approximately 1.1 mm. This base element size in the bulk flow region was chosen to ensure convergence of the simulations, per previous studies by Bartol et al. (2024). The total number of polyhedral cells resulted in 60,204 and 177,802 mesh vertices. The total volume of the lower respiratory tract model of the subject was 19.82 mL.

Within both the trachea and the primary bronchi, large eddies and flow separation arise from the abrupt change in direction at the carina ridge. These large-scale turbulent structures are captured by the k-ω Shear-Stress Transport (SST) Langtry–Menter (LM) RANS turbulence model. This four-equation model resolves transitional and fully developed turbulence and captures relaminarization in distal bronchi. Although detailed information on boundary conditions, choice of turbulence model, and fluid-particle coupling can be found in previous work by Bartol et al. (2024), the following description provides a summary of the boundary conditions used for the model.

At the outlets, the pressure was set to a fixed value, while zero-gradient conditions were applied at the inlets and walls. Turbulence inlet specification was computed with the usual isotropic estimate for the kinetic energy, $k=\frac{3}{2}{\left(I\left|u_{\text{ref}}\right|\right)}^{2}$. Because at heavy-exercise conditions, a medium level of turbulence is expected, the intensity I was set to 4%, and the reference speed $u_{\text{ref}}$ was the peak inhalation velocity at the inlet for the particular airflow used. The specific dissipation rate ω is then set with $\omega =k^{1/2}/\left(C_{\mu }^{1/4}L\right)$, using the SST constant $C_{\mu }=0.09$ and a length scale L equal to the hydraulic diameter of the inlet. At the outlets, both k and ω were set to the condition of zero pressure gradients.

The *All* y+ *wall treatment* wall function was used in STAR-CCM+, which blends wall functions and near-wall resolution models depending on the local $y^{+}$ value. In our simulation, the $y^{+}$ values ranged from 0 to 5, falling within the viscous sublayer.

The intermittency γ is set to 1.0 at the inlet, assuming fully turbulent conditions, and uses zero gradients elsewhere in the regions. Lastly, the transition momentum thickness Reynolds number $Re_{\theta }$ is computed from the local turbulence intensity Tu with a more complex piece-wise correlation. Walls and outlets again were set with zero gradients for both γ and $Re_{\theta }$.

A total of 105,492 particles were injected into the system using 20 randomized injector points located on the inlet surface, corresponding to the trachea inlet. Of the total original particles injected, only 17,839 deposited to the walls.

Three boundary conditions were implemented for the volume flow inlet: a baseline flow rate of ${\dot{v}}_{1}=50\;\text{L}/\text{min}$, followed by configurations with 20% higher $\left({\dot{v}}_{2}=60\;\text{L}/\text{min}\right)$ and 20% lower $\left({\dot{v}}_{3}=40\;\text{L}/\text{min}\right)$ flow rates. An additional simulation with 10% increased flow $\left({\dot{v}}_{4}=55\;\text{L}/\text{min}\right)$ was performed to validate the interpolation capabilities of the Koopman operator.

### 2.2. Data arrangement

For particle tracking, snapshots of particle positions for the baseline simulation were stacked vertically to form a third-ordered tensor $X_{\text{particles}}\in R^{\left(3\times N_{p}\times N_{t}\right)}$, where:

- 3 is the number of spatial coordinates (x,y,z);
- $N_{p}$ is the number of particles for the DMD study $\left(N_{p}=17839\right)$; and
- $N_{t}$ is the number of uniform timesteps (m = 144).

Only the trajectories of particles that deposited to the walls at their last position were used; particles that were inhaled and then exhaled were omitted from the tensor definition.

Similarly, the fluid velocity field was organized into a tensor $X_{\text{fluid}}\in R^{\left(3\times N_{n}\times N_{t}\right)}$, where:

- 3 is the number of velocity components (u,v,w);
- $N_{n}$ is the number of mesh nodes $\left(N_{n}=177802\right)$; and
- $N_{t}$ is the number of uniform timesteps (m = 144).

### 2.3. Exact dynamic mode decomposition

The exact DMD algorithm from Kutz and Brunton (2016) was applied to both datasets, $X_{\text{fluid}}$ and $X_{\text{particles}}$.

For the decomposition of the particles, the lagged $\left(X_{\text{particles}}^{-}\right)$ and shifted $\left(X_{\text{particles}}^{+}\right)$ tensors were constructed by arranging each state vector in chronological order. Each vector contains the three spatial coordinates of each particle at time i, represented here as $x_{i}\in R^{3\times N_{p}};x_{i}=\left(x_{i},y_{i},z_{i}\right)$, with each column corresponding to a different particle:

(3) $$X_{\text{particles}}^{-}=\left[x_{1},x_{2},\ldots ,x_{m-1}\right],\;X_{\text{particles}}^{+}=\left[x_{2},x_{3},\ldots ,x_{m}\right]$$

Here m is the number of snapshots taken for the simulation, which for all purposes of this study was m=144.

For the $X_{\text{fluid}\left({\dot{v}}_{j}\right)}$ tensors corresponding to each of the j-th breathing flow rates $\left({\dot{v}}_{j}\in \left\{{\dot{v}}_{1},{\dot{v}}_{2},{\dot{v}}_{3},{\dot{v}}_{4}\right\}\right)$, where the index j refers to the breathing flow rate used for the corresponding simulation. The data is arranged as $X_{\text{fluid}\left({\dot{v}}_{j}\right)}=\left[x_{1}^{j},x_{2}^{j},\ldots ,x_{m}^{j}\right]$, from that structure, the following tensors were then constructed as:

(4) $$X_{\text{fluid}\left({\dot{v}}_{j}\right)}^{-}=\left[x_{1}^{j},x_{2}^{j},\ldots ,x_{m-1}^{j}\right],\;X_{\text{fluid}\left({\dot{v}}_{j}\right)}^{+}=\left[x_{2}^{j},x_{3}^{j},\ldots ,x_{m}^{j}\right]$$

before applying DMD, each snapshot $x_{i}\in R^{3\times N_{p}}$ was reshaped into a column vector $x_{i}\in R^{3N_{p}}$, forming the snapshot matrix

$$X_{\text{particles}}^{-}\in R^{\left(3N_{p}\right)\times \left(N_{t}-1\right)}$$

(and similarly for $X_{\text{fluid}}$). The lagged tensor is subsequently decomposed using SVD.

(5) $$X_{\text{particles}}^{-}=U\Sigma V^{T}$$

(6) $$X_{\text{fluid}\left({\dot{v}}_{j}\right)}^{-}=U^{j}\Sigma^{j}{\left(V^{j}\right)}^{T}$$

The matrix $U\in R^{n\times m}$ (and its transpose $U^{T}\in R^{m\times n}$) contains the spatial orthonormal POD modes of the simulation. The diagonal matrix $\Sigma \in R^{m\times m}$ holds the singular values, which represent the energy captured by each corresponding mode. The matrix $V\in R^{m\times m}$ (and its transpose $V^{T}\in R^{m\times m}$) contains the temporal coefficients that describe how the spatial modes in U are weighted and combined over time.

The DMD operator $A\in R^{n\times n}$ with rank(A)=m≪n is approximated as $\tilde{A}\in R^{r\times r}$ by projecting it onto the subspace spanned by the truncated POD modes $U_{r}\in R^{n\times r}$, retaining only a subset of r≤m=144 dominant modes. The derivation proceeds as follows:

(7) $$\tilde{A}=U_{r}^{T}A\;U_{r}$$

(8) $$X^{+}=AX^{-}=AU\Sigma V^{T}$$

(9) $$A=X^{+}V\Sigma^{-1}U^{T}$$

(10) $$U^{T}U_{r}={\left[\begin{array}{r} I_{r}\\ 0 \end{array}\right]}_{n\times r}$$

(11) $$\tilde{A}=U_{r}^{T}X^{+}V_{r}\Sigma_{r}^{-1}$$

Where $I_{r}\in R^{r\times r},0\in R^{(n-r)\times r},V_{r}\in R^{m\times r}$, and $\Sigma_{r}\in R^{r\times r}$. Combining these components yields:

(12) $${\tilde{A}}_{\text{particles}}=U_{r}^{T}X_{\text{particles}}^{+}V_{r}\Sigma_{r}^{-1}\;{\tilde{A}}_{\text{fluid}\left({\dot{v}}_{j}\right)}={\left(U_{r}^{j}\right)}^{T}X_{\text{fluid}\left({\dot{v}}_{j}\right)}^{+}V_{r}^{j}{\left(\Sigma_{r}^{j}\right)}^{-1}$$

The DMD modes $\Phi \in R^{n\times r}$ and eigenpairs $\Lambda_{r}\in R^{r\times r}$ (eigenvalues) and $W_{r}\in R^{r\times r}$ (eigenvectors) are obtained by performing eigen-decomposition on each of the $\tilde{A}$:

(13) $${\tilde{A}}_{\text{fluid}\left({\dot{v}}_{j}\right)}W_{r}^{j}=W_{r}^{j}\Lambda_{r}^{j}$$

(14) $$\Phi_{\text{fluid}\left({\dot{v}}_{j}\right)}=X_{\text{fluid}\left({\dot{v}}_{j}\right)}^{+}V_{r}^{j}{\left(\Sigma_{r}^{j}\right)}^{-1}W_{r}^{j}$$

(15) $${\tilde{A}}_{\text{particles}}W_{r}=W_{r}\Lambda_{r}$$

(16) $$\Phi_{\text{particles}}=X_{\text{particles}}^{+}V_{r}\Sigma_{r}^{-1}W_{r}$$

An approximation of the original data, $X_{\text{reconstructed}}(t)$, can be reconstructed for a dimensionless time t/Δt, where Δt=0.015s represents the discrete timestep used in both the simulation and the DMD formulation, the last 16 frames of the 2.4s breathing cycle were discarded as the fluid field was nearly zero throughout the geometry, causing numerical instability in the DMD. The reconstruction is obtained by computing the initial amplitudes $b_{0}\in R^{r\times 1}$ using the pseudoinverse of the previously computed DMD modes Φ.


(17) $$b_{0}=\Phi^{†}x_{1}$$

(18) $$X_{\text{reconstructed}}(t)=\Phi \cdot \Lambda^{t/\Delta t}\cdot b_{0}$$

This reconstruction is made for each of the simulations by choosing the appropriate Φ,Λ and $b_{0}$.

### 2.4. Koopman operator interpolation

A linear combination of Koopman operators (Huhn et al., 2023) was performed for flow rate interpolation between boundary conditions. Given two trained operators ${\tilde{A}}_{1}$ and ${\tilde{A}}_{2}$ with corresponding breathing flow rates ${\dot{v}}_{1}$ and ${\dot{v}}_{2}$, the interpolated operator ${\tilde{A}}_{\theta }$ for an intermediate flow rate ${\dot{v}}_{\theta }$ is:

(19) $${\tilde{A}}_{\theta }=\theta {\tilde{A}}_{1}+(1-\theta ){\tilde{A}}_{2}$$

where θ∈[0,1] is the interpolation weight for an intermediate ${\dot{v}}_{\theta }$ determined by:

(20) $$\theta =\frac{{\dot{v}}_{\theta }-{\dot{v}}_{2}}{{\dot{v}}_{1}-{\dot{v}}_{2}}$$

Then, performing the eigen-decomposition of the interpolated Koopman operator to obtain the eigenpair.


(21) $${\tilde{A}}_{\theta }W^{\theta }=\Lambda^{\theta }W^{\theta }$$

The POD modes can be interpolated $\left(U^{\theta }\right)$ to compute the interpolated DMD modes $\left(\Phi_{\theta }\right)$ by projecting onto the POD modes since the $X_{\text{fluid}(\theta )}^{+}$ is not available to compute the modes via the exact way as in Eq. (14), and thus computed as follows:

(22) $$U_{\theta }=\theta U^{1}+(1-\theta )U^{2}$$

(23) $$\Phi_{\theta }=U^{\theta }W^{\theta }$$

Finally, the amplitudes are interpolated as: $\left(b_{0}^{\theta }\right)$ as:

(24) $$b_{0}^{\theta }=\theta b_{0}^{1}+(1-\theta )b_{0}^{2}$$

The reconstructed field $X_{\text{reconstructed}}^{\theta }(t)$ is then computed using Equation (18) with $\Phi_{\theta },\Lambda^{\theta }$ and $b_{0}^{\theta }$.

All the computations described above follow an order that can be summarized in the following algorithm:

**Algorithm 1:** Parametric DMD algorithm via reduced (discrete) Koopman operator interpolation (Huhn et al., 2023)

1.	Solve the fluid field for each of the $N_{S}$ breathing flow rates ${\left\{{\dot{v}}_{j}\right\}}_{j=1}^{N_{S}}$ and collect temporal snapshots.
2.	Divide each matrix of snapshots into a shifted matrix $X_{\text{fluid}\left({\dot{v}}_{j}\right)}^{+}$ and a lagged matrix $X_{\text{fluid}\left({\dot{v}}_{j}\right)}^{-}$.
3.	Perform SVD of $X_{\text{fluid}\left({\dot{v}}_{j}\right)}^{-}:X_{\text{fluid}\left({\dot{v}}_{j}\right)}^{-}=U^{j}\Sigma^{j}{\left(V^{j}\right)}^{T}$, for $1\leq j\leq N_{S}$.
4.	Retain r modes and compute the reduced Koopman operator $\tilde{A}$ for each of the training breathing flow rates $\left({\dot{v}}_{j}\right)$, see Eq. (12).
5.	Interpolate the Koopman operator ${\tilde{A}}_{\theta }$ using the respective operators ${\tilde{A}}_{\text{fluid}\left({\dot{v}}_{1}\right)}$ and ${\tilde{A}}_{\text{fluid}\left({\dot{v}}_{2}\right)}$ of the closest breathing flow rates $\left({\dot{v}}_{1},{\dot{v}}_{2}\right)$ to the breathing flow rate of interest $\left({\dot{v}}_{\theta }:{\dot{v}}_{1}<{\dot{v}}_{\theta }<{\dot{v}}_{2}\right)$, see Eq. (19).
6.	Perform the eigen-decomposition of ${\tilde{A}}_{\theta }$ to obtain the reduced eigen-pair $\left(\Lambda^{\theta },W^{\theta }\right)$, see Eq. (21).
7.	Interpolate the POD modes $U^{\theta }$ using the respective $U^{1}$ and $U^{2}$ of the closest breathing flow rates $\left({\dot{v}}_{1},{\dot{v}}_{2}\right)$ to the breathing flow rate of interest $\left({\dot{v}}_{\theta }:{\dot{v}}_{1}<{\dot{v}}_{\theta }<{\dot{v}}_{2}\right)$, see Eq. (22).
8.	Construct the DMD modes $\Phi_{\theta }=U^{\theta }W^{\theta }$, see Eq. (23).
9.	Interpolate the initial amplitudes $b_{0}^{\theta }$ using the respective $b_{0}^{1}$ and $b_{0}^{2}$ of the closest breathing flow rates $\left({\dot{v}}_{1},{\dot{v}}_{2}\right)$ to the breathing flow rate of interest $\left({\dot{v}}_{\theta }:{\dot{v}}_{1}<{\dot{v}}_{\theta }<{\dot{v}}_{2}\right)$, see Eq.(24).
10.	Reconstruct the full-state solution $X_{\text{reconstructed}}^{\theta }(t)$, see Eq. (18).

### 2.5. Performance evaluation

To evaluate the performance of the DMD reconstruction, three quantitative metrics were used: (1) Mean Relative Error (MRE), to assess the general precision of fluid field reconstructions; (2) the Bhattacharyya correlation coefficient, which measures the similarity between two probability distributions by quantifying their overlap (where a value of 1 indicates a perfect match and 0 indicates completely dissimilar distributions). This metric was used to compare the reconstructed and ground-truth particle deposition distributions; and (3) the maximum local relative error, to identify the worst-case deviations in spatial precision.

These metrics were applied in each phase of the study to quantify the fidelity of the reconstruction. The Bhattacharyya correlation was computed as follows:

(25) $$\text{Bhattacharyya}\;\text{Correlation}\left(D_{CFD},D_{DMD}\right)=\sum_{r\in [0,R]}\sqrt{D_{CFD}(r)\cdot D_{DMD}(r)}$$

Where $D_{CFD}(r)$ and $D_{DMD}(r)$ are the particle marginal deposition distributions at position r, obtained by CFPD simulations and DMD reconstruction respectively.

The CFD and DMD data matrices $X_{\text{CFD}},X_{\text{DMD}}\in R^{3\times N_{n}\times N_{t}}$ contain the velocity components (u,v,w) across all $N_{n}$ mesh nodes and $N_{t}$ time steps, where each column corresponds to one temporal snapshot. That is,

$$X_{\text{CFD}}=\left[\begin{array}{cccc} | & | & & |\\ x_{1}^{\text{CFD}} & x_{2}^{\text{CFD}} & \cdots & x_{N_{t}}^{\text{CFD}}\\ | & | & & | \end{array}\right],\;X_{\text{DMD}}=\left[\begin{array}{cccc} | & | & & |\\ x_{1}^{\text{DMD}} & x_{2}^{\text{DMD}} & \cdots & x_{N_{t}}^{\text{DMD}}\\ | & | & & | \end{array}\right],$$

where $x_{k}^{\text{CFD}},x_{k}^{\text{DMD}}\in R^{3\times N_{n}}$ are the CFD and DMD state vectors at the kth time step.

The relative reconstruction error for each snapshot was computed as

(26) $$\epsilon_{k}=\frac{{\left\|x_{k}^{\text{CFD}}-x_{k}^{\text{DMD}}\right\|}_{2}}{{\left\|x_{k}^{\text{CFD}}\right\|}_{2}}\times 100\%,$$

where each $\epsilon_{k}$ quantifies the relative $L_{2}$-error between the DMD reconstruction and the ground-truth CFD field at time step $t_{k}$.

The mean relative error reported in Table 1 corresponds to the arithmetic average of the individual snapshot errors:

(27) $$\text{MRE}=\frac{1}{N_{t}}\sum_{k=1}^{N_{t}}\epsilon_{k}.$$

Finally, the maximum relative error in all of time-steps, was calculated as:

(28) $$\epsilon_{\text{max}}=max(\epsilon )$$

## 3. Results

The results are organized according to the three phases of the study: Lagrangian particle reconstruction, fluid field reconstruction, and interpolation using the Koopman operator. For each phase, performance was assessed using the defined quantitative metrics, MRE, Bhattacharyya correlation, and local relative error, alongside qualitative visualization of DMD-reconstructed fields. Additionally, computational efficiency was compared between DMD-based and full CFD simulations.

### 3.1. Phase I results: Lagrangian reconstruction

**Fig. 2.**
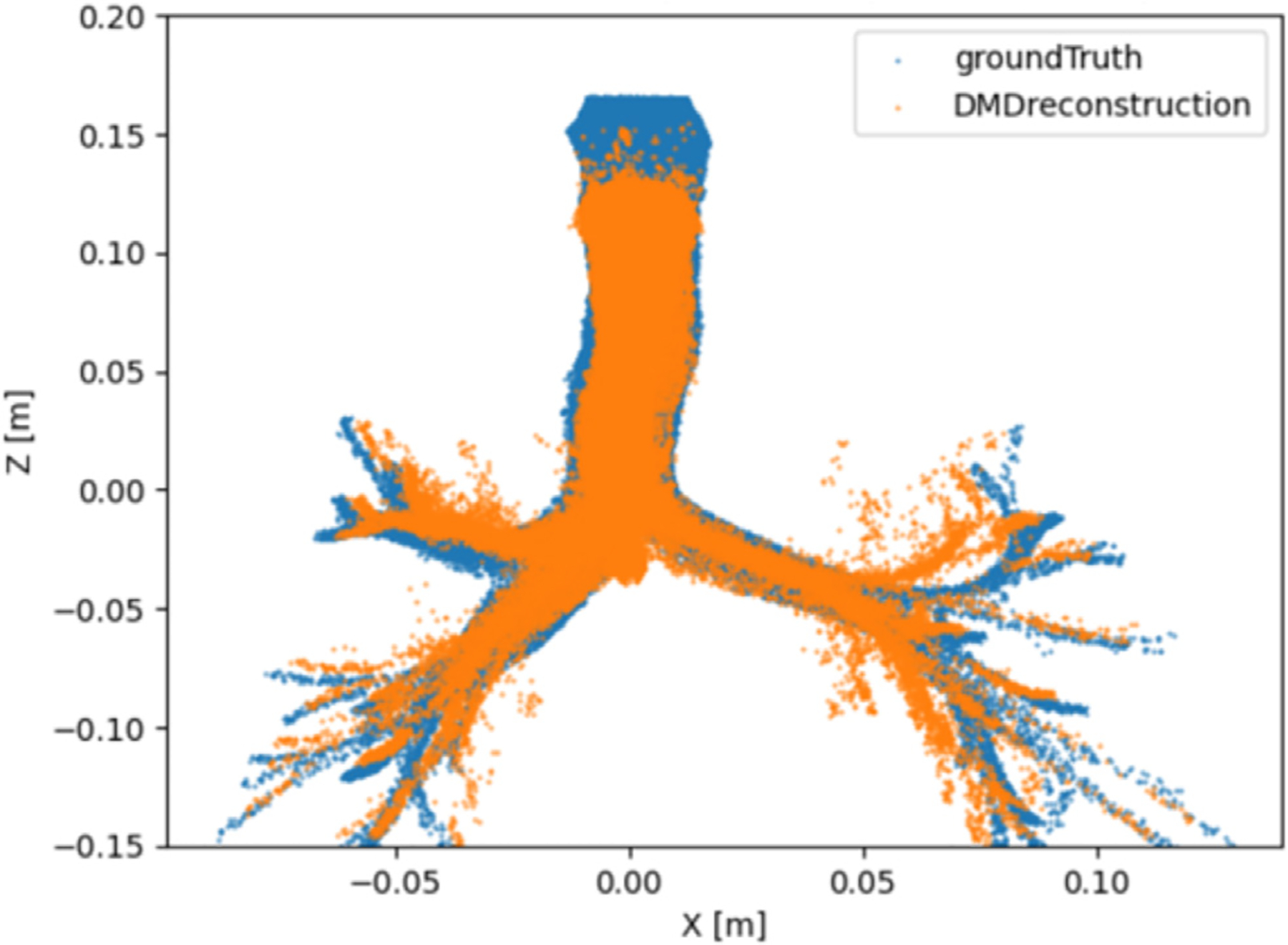
Particle trajectory comparison in Cartesian coordinates. While both ground truth (blue) and DMD reconstruction (orange) maintain similar path morphology, visible divergence occurs in bronchial region. The reconstruction preserves directional trends but shows slight phase offsets, particularly where trajectories curve sharply.

**Fig. 3.**
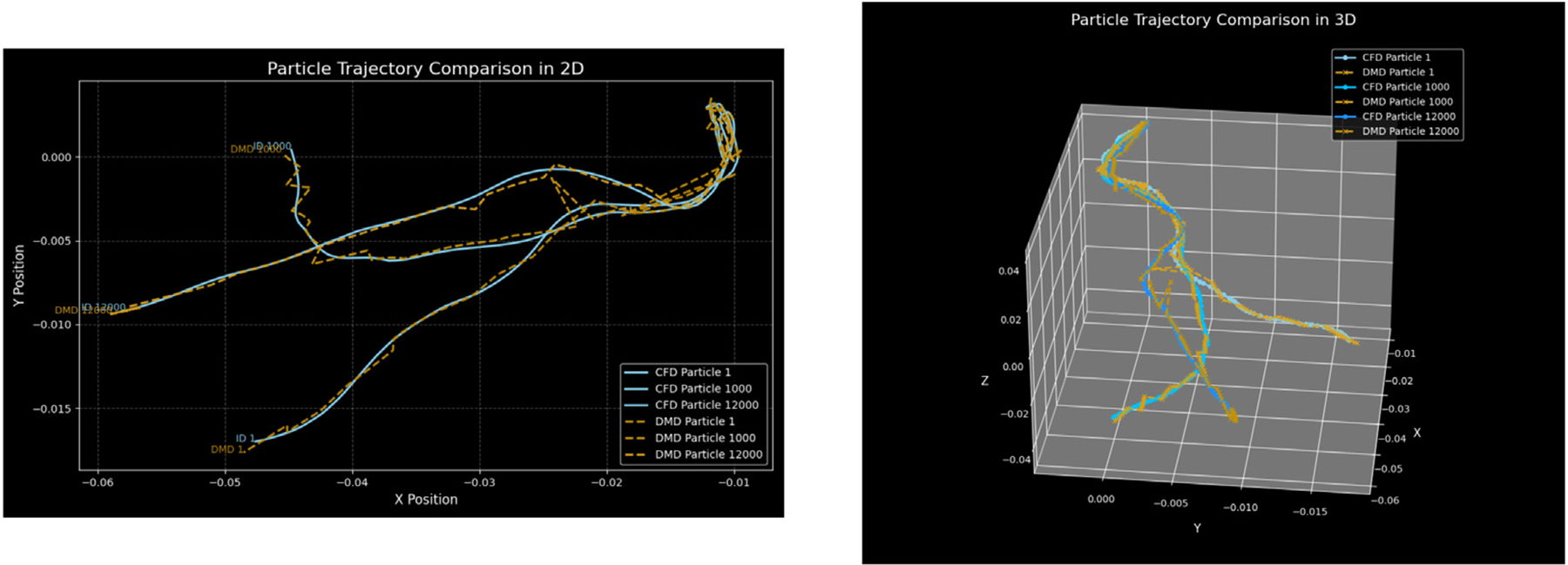
Comparison between CFD-simulated and DMD-reconstructed particle trajectories. For all trajectories, the root mean squared error (RMSE) between the ground truth and the predicted trajectories was < 1%. **Left:** 2D projection (XY plane) of individual particle trajectories, showing the deviations between the original CFD paths and the corresponding DMD reconstructions for three particles. Right: 3D view of the same trajectories, illustrating how the DMD model approximates the full spatial paths.

**Fig. 4.**
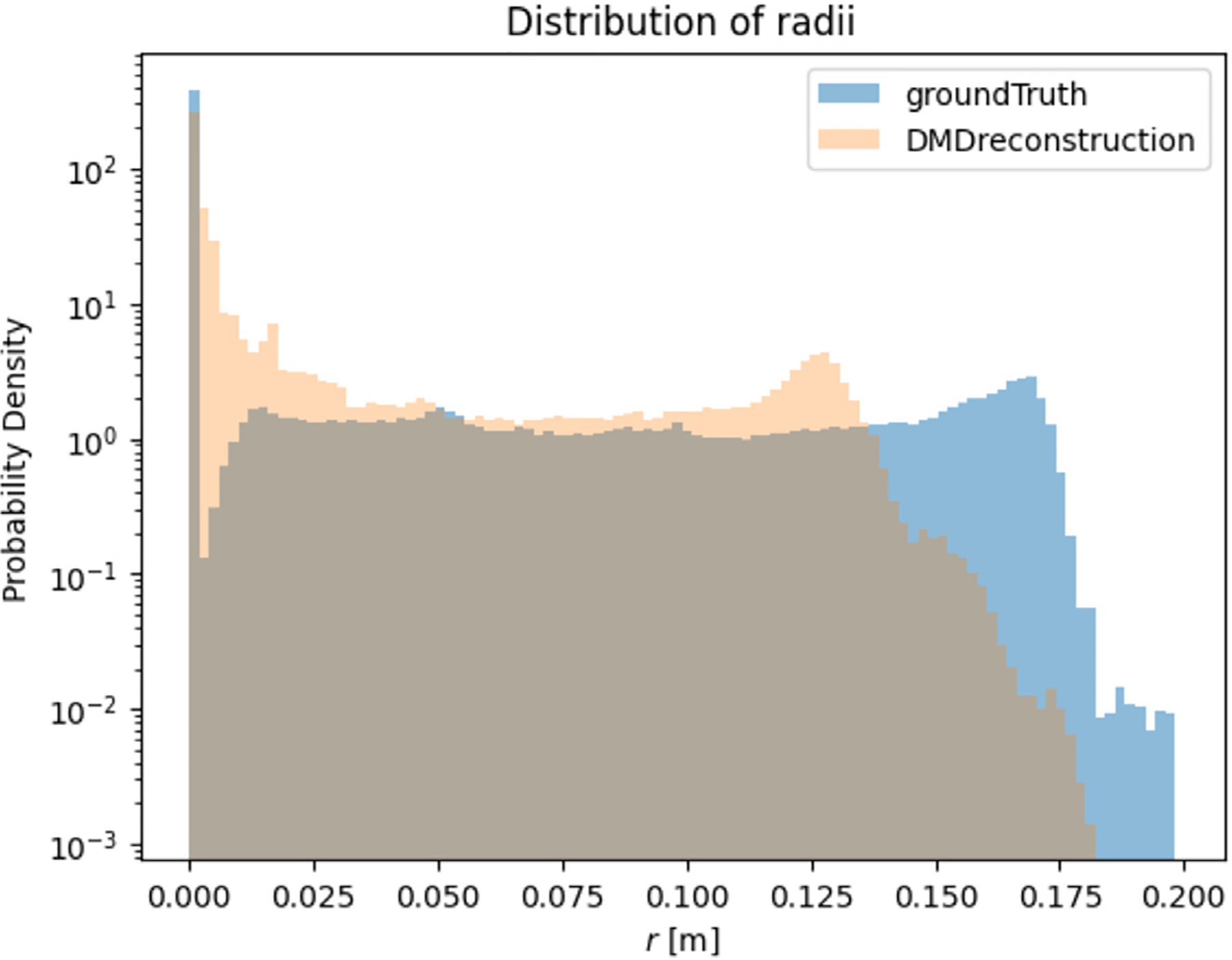
Marginal probability density comparison of radial particle deposition. DMD reconstruction (orange) closely matches the ground truth (solid blue) distribution across all positions. Minor discrepancies appear away and very close to the Carina (center of the system) where the reconstruction slightly underestimates particle density, achieving a Bhattacharyya correlation coefficient of 85%.

For each dataset, N particle trajectories were tracked until they impinged on the wall, N=17839 particles. The radial position $r_{j}$ of every deposited particle j was recorded. In Fig. 2, a visual comparison between the CFPD (blue) and DMD (orange) predicted trajectories was presented. The Lagrangian reconstruction analysis revealed two key aspects of DMD performance. Spatial tracking, as in Fig. 2, demonstrated that DMD successfully captured the overall trajectory patterns but accumulated positional errors in regions of complex flow, evidenced by the diverging paths in the smaller bronchi branches. The directional consistency suggested that phase relationships were preserved despite local deviations. It is important to note that the DMD model does not explicitly evaluate whether a particle remains within or exits the geometric domain, but instead reconstructs trajectories based on the spatial and temporal information captured in the DMD modes. The geometry is implicitly reflected in the particle paths, rather than being directly encoded or constrained. As a result, some deviations from the original trajectories. To observe this phenomenon, three individual particle trajectories sampled from the original tensor, were reconstructed using DMD, as shown in Fig. 3. The results show that the reconstructed paths may extend beyond the original geometry, since they do not follow the exact particle trajectory at each time step. In Fig. 4, a histogram with uniform bin width Δr=0.01R was constructed and normalized:

(29) $$p_{i}=\frac{n_{i}}{N\;\Delta r},\;i=1,\ldots ,\frac{R}{\Delta r},$$

where $n_{i}$ is the count in bin i.

The same binning and normalization were applied to (a) the Euler–Lagrange CFD data and (b) the DMD-predicted particle field, which was sampled with identical binning resolution.

The Position (r) axis in Fig. 4, where r∈[0,R] denotes the radial distance from the carina–centroid axis. The marginal probability-density function (PDF) p(r) is defined such that,

(30) $$\int_{0}^{R}p\left(r\right)\;dr=1,$$

i.e. $p\left(r\right)dr$ gives the probability that a particle deposits between r and r+dr. The probability density p(r) can take values ≫ 1, meaning high particle concentration in narrow bins or ≪ 1, meaning low particle concentration in those bins.

Since only particles that ultimately adhered to the airway walls were considered in the analysis, all reconstructed trajectories correspond to deposited particles, with their final positions indicating their deposition sites. In the study, no particles that had escaped through the outlets or inlet were considered. Another analysis focused on how well the DMD model captured the particle distribution based on a Euclidean distance metric, Eq. (31), with respect to a fixed point ($\vec{p_{0}}=\left(x_{0},y_{0},z_{0}\right)$ located in the Carina) used for both the ground truth particle simulation and the DMD reconstruction. The distribution analysis, shown in Fig. 4, demonstrated a strong agreement with the ground truth, with DMD reproducing the overall deposition density profile in all radial positions with a Bhattacharyya correlation coefficient of 85%. The presented method captured peak deposition in the carinal ridge, while showing slight underprediction in the smaller bronchi or more distant regions, likely due to the fact that particles traveling farther down the airway follow more convoluted trajectories. In conclusion, the presented DMD approach prove more effective for predicting statistical deposition patterns than for tracking individual particle trajectories, completing the reconstruction in under 7 min using full-ranked Koopman operators (r=144), whereas the ground truth simulation required approximately 60 h to model a complete 2.4-second breathing cycle.


(31) $$\text{Radial}\;\text{distance}[m]=\sqrt{{\left(x-x_{0}\right)}^{2}+{\left(y-y_{0}\right)}^{2}+{\left(z-z_{0}\right)}^{2}}$$

### 3.2. Phase II results: Fluid field reconstruction

**Fig. 5.**
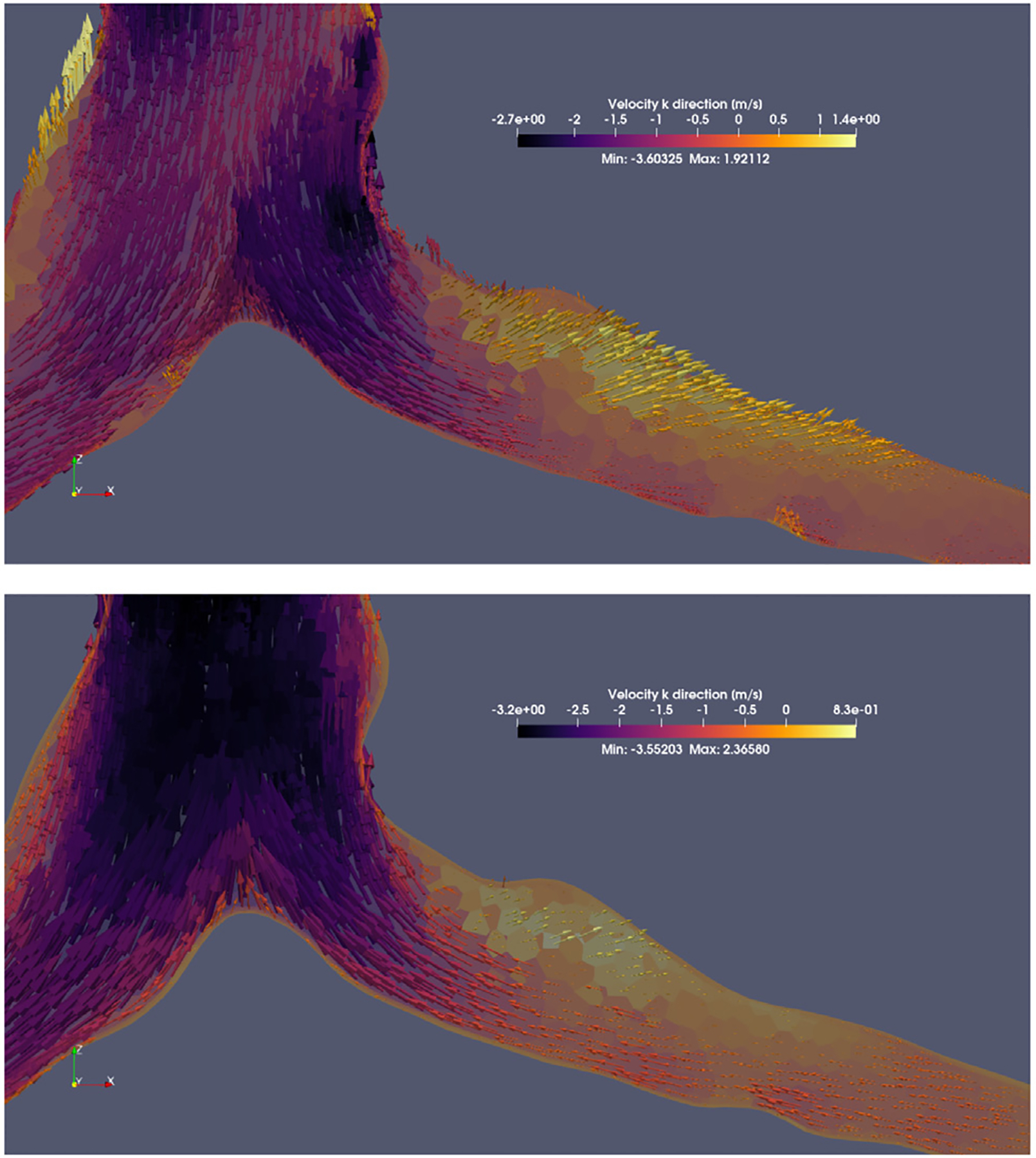
Recirculation zones identified in both the DMD reconstruction and the CFD simulation. Vortices are visible as arrows or fluid parcels flow in opposing directions within regions of flow separation. A noticeable damping of the maximum velocity is also observed in the maximum velocity magnitude throughout the entire geometry (Max: 2.36 m/s for CFD and Max: 1.92 m/s for DMD). **Top**: Recirculation pattern from DMD reconstruction. **Bottom**: Recirculation pattern from CFD simulation.

**Fig. 6.**
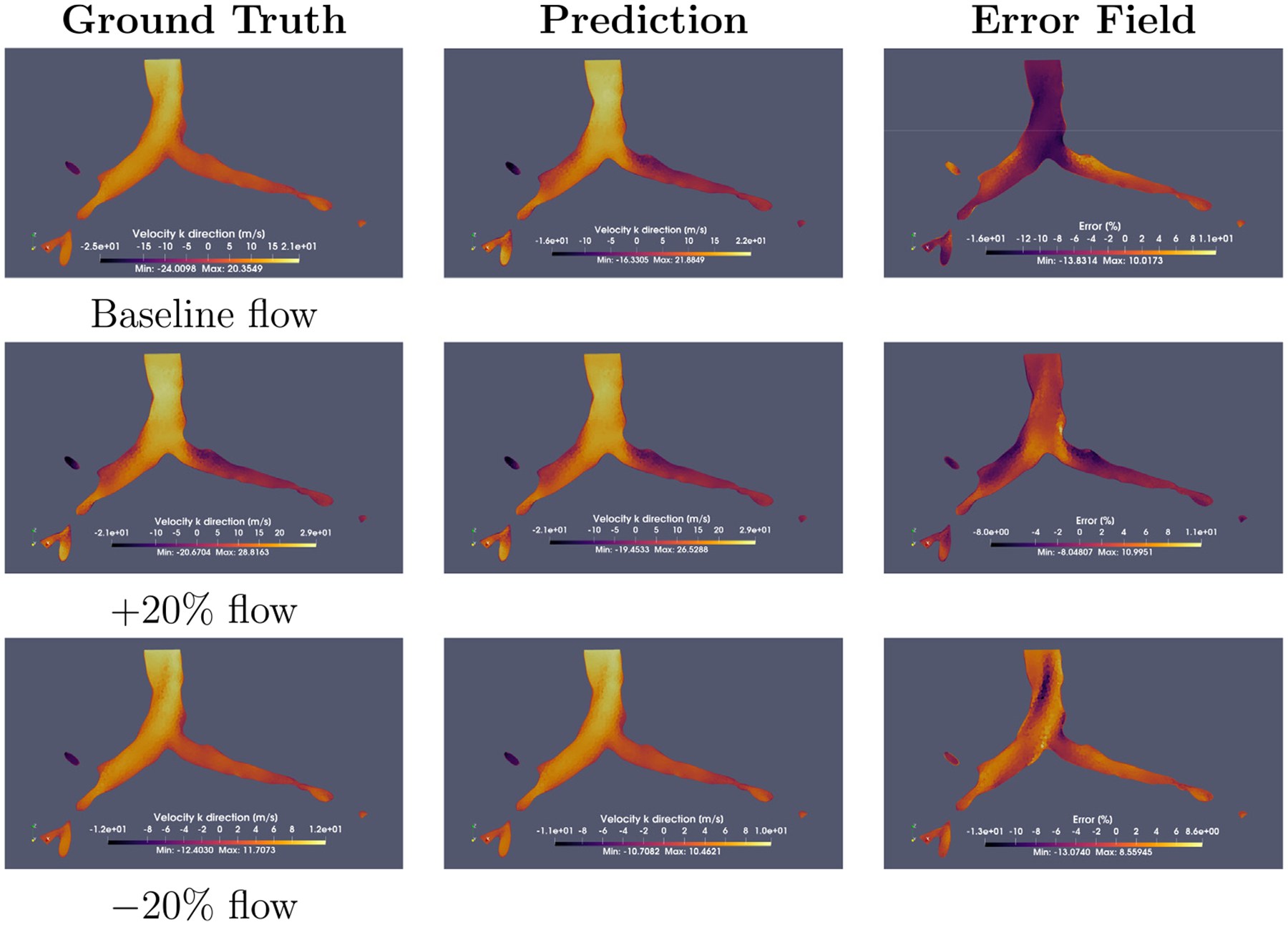
Worst-case reconstruction error analysis across flow conditions. Columns show (left) high-fidelity simulation, (middle) DMD prediction, and (right) relative error map. Error concentrations appear near bifurcations but remain bounded, preserving large-scale flow structures. All cases maintain consistent recirculation zones (dark purple areas).

The fluid field reconstruction phase demonstrated DMD’s ability to capture the main flow features under varying inlet conditions. While DMD successfully resolved recirculation zones and dominant flow patterns (Fig. 5), maximum errors arose in regions where flow separation occurs because of the abrupt change in the geometry; these are typically locations with steep velocity gradients (e.g., bronchial branching). A worst-case analysis is presented in Fig. 6, revealed localized discrepancies near flow bifurcations (flow-splitting regions), where error magnitudes scaled with flow complexity. It is noteworthy that errors never exceeded 15% at any single-cell level across all inlet flow conditions.

Notably, although DMD preserved main flow features, such as flow separation in high-curvature regions, vortical recirculation, and near-stagnant regions, across all volume flow rates, it also introduced progressive damping of the overall kinetic energy. This smoothing effect, observed consistently across cases, probably originated in the rank reduction step in DMD, where the less dominant modes were truncated to increase computational efficiency, resulting in a total reconstruction time of ~10 min, including file generation.

### 3.3. Phase III results: Koopman interpolation

**Fig. 7.**
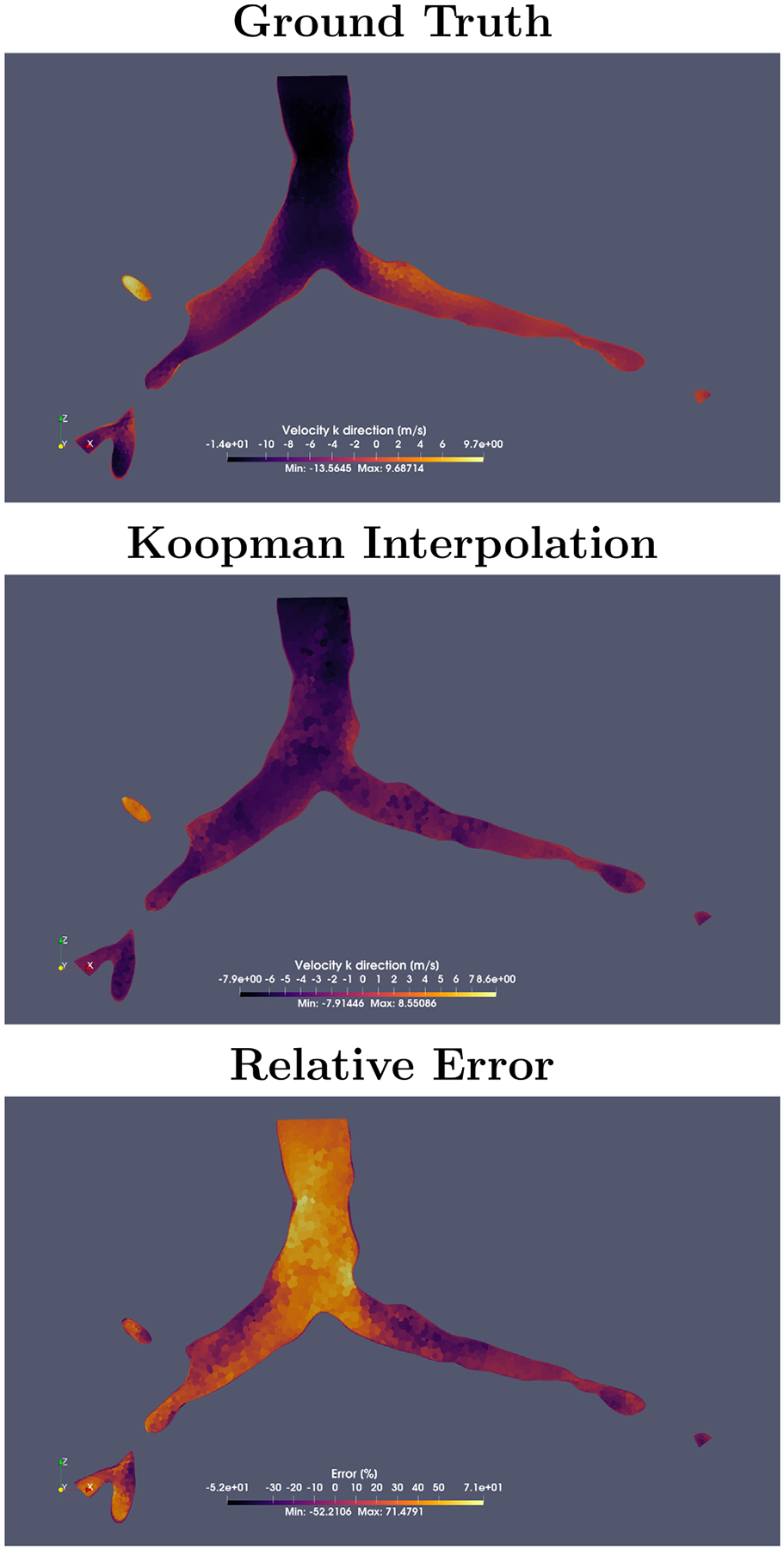
Interpolation performance for +10% volume flow case. **Top:** High-fidelity simulation showing ground truth. **Center**: Koopman-based reconstruction preserves large-scale flow patterns but loses small-scale flow features. **Bottom**: Error field reveals maximum discrepancies in shear layers and tracheal region, with relative errors reaching more than 70% in high-velocity gradients.

The Koopman operator interpolation highlighted the challenges of projecting nonlinear system dynamics onto a finite-dimensional subspace. By combining modes from 100% and 120% input volume flow rate simulations, the method preserved topological features of the flow field, such as the primary recirculation zones in the modeled parts of the tracheobronchial region (G0 to G5), including the yellow region in Fig. 7, and bulk flow direction. However, the large interpolation interval (20%) struggled to resolve the geometric complexity of the domain, leading to two critical issues: (1) Progressive damping of the mean velocity (loss of kinetic energy fidelity) and (2) inaccurate reconstruction of recirculation regions in bronchial flow-splitting zones (bifurcations). While the mean velocity profile maintained correlation, localized errors concentrated in regions sensitive to volume flow variations, notably bifurcation zones and boundary layer near walls. This suggested that while Koopman interpolation preserved essential nonlinear behavior through its infinite-dimensional projection, practical applications in respiratory flows require training data with smaller parameter intervals to resolve geometrically induced flow instabilities. This necessity reduces the original advantage of the method of reducing computational burden through sparse sampling, as a finer parameter resolution requires more expensive simulations due to a smaller time step.

Table 1 provides a summary of the maximum absolute relative local error and the MRE in all snapshots for each of the conditions studied previously together with the interpolation case and a localized measurement of the error on the walls and outlets of the model. All reconstructions were performed using full-ranked (r = 144) Koopman operators.

**Fig. 8.**
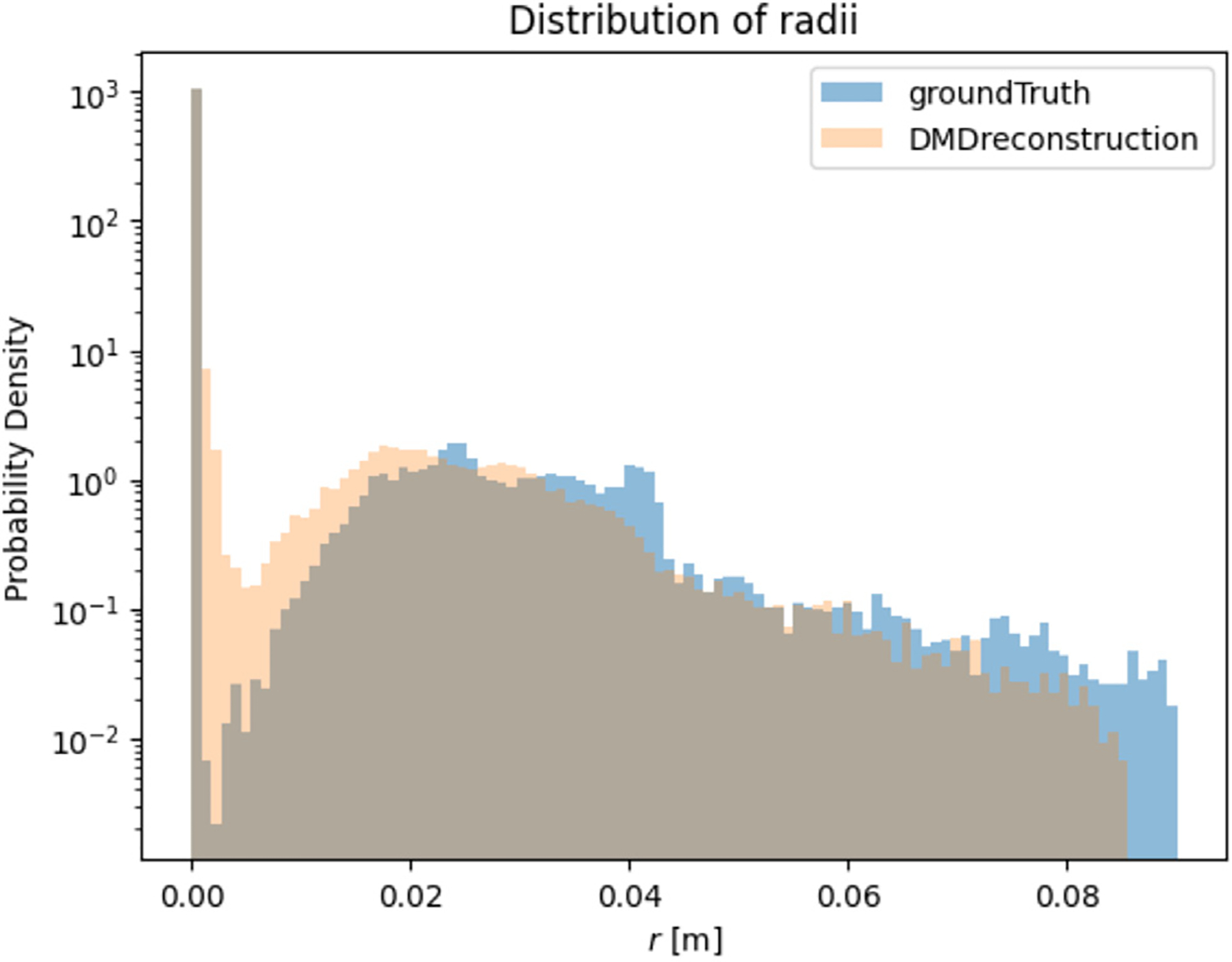
Marginal probability density comparison of radial particle deposition. The DMD reconstruction (orange) closely matches the ground truth distribution (solid blue) across all radial positions relative to the carina origin, with a Bhattacharyya correlation coefficient of 80%. Discrepancies are most notable at the smallest distance range, indicating differences near the carina similar to those observed in Fig. 4.

The interpolation scheme was also applied to the deposition distributions from simulations at 100% and 120% input volume flow rates. Its performance was comparable to the direct reconstruction results shown in Fig. 4, achieving a Bhattacharyya correlation coefficient of 80%. The corresponding results are presented in Fig. 8.

## 4. Discussion

This study investigated the application of DMD in fluid and particle simulations as a reduced-order model, i.e., as a framework in which simulations can be reconstructed at a fraction of the computational cost.

In the first part of this study (Section 3.1), the sensitivity of DMD to trajectory reconstructions was demonstrated. The distribution of particles deposited on the walls at the end of the simulation is strongly correlated with the ground truth, although the exact positions and trajectories are not well reconstructed. This behavior is likely attributable to the lack of coupling among particles, despite their overall path similarity. Each row of the input tensor corresponds to an individual particle and is treated independently, without enforcing inter-particle constraints. As shown in Fig. 3, the reconstructed trajectories diverge from the ground-truth paths, and in Fig. 2, several particles are observed outside the boundaries of the original three-dimensional geometry. In contrast, when simulating fluid fields (Sections 3.2 and 3.3), the momentum equations tightly couple all the nodes (rows) of the input matrix, thereby enabling the DMD framework to better capture the system dynamics and achieve a lower reconstruction error.

Because training excluded non-deposited trajectories, the DMD cannot predict whether a particle exits the domain or remains in suspension; it only localizes deposited particles. Estimation of deposition probability/fraction by size or release location should be provided by a complementary model (e.g., ICRP/MPPD or a dedicated classifier), after which our DMD yields spatial localization for the predicted depositors.

Most data-driven approaches are unbounded by governing equations, making them prone to divergence when the input lies outside the vicinity of the training set. However, the results in Section 3.3 show that DMD produces outcomes consistent with neighboring datasets. A 10% variation in the initial conditions results in an increase in mean reconstruction error by comparable margin, with localized errors increasing by nearly sevenfold.

In the study by Habibi et al. (2020), the authors applied DMD to a pulsatile blood flow system (an oscillating system similar to a breathing cycle). However, they imposed a boundary condition at the pressure inlet consisting of a sum of multiple overlapping complex exponentials (i.e., sines and cosines), making their system similarly intricate. They validated their results against the Womersley solution, which served as the ground truth. Notably, (Habibi et al., 2020) found that reducing the system to only 10 modes resulted in an error of less than 10%, comparable to the error obtained in this study, as shown in Table 1, with the exception of the interpolation case, which involves additional numerical error sources.

In the work by Jing et al. (2024), the authors concluded that DMD is a particularly suitable framework for reconstructing respiratory flows, as it captures single-frequency dynamics. This ability allows DMD to isolate the components comprising the entire simulation and to offer a different perspective on particle transport and deposition. These conclusions are consistent with the results presented in Sections 3.1 and 3.2. Together, these findings reinforce the potential for reduced-order dynamic mode representations to approximate high-fidelity simulations in inhalation dosimetry applications, while also underscoring the importance of spatial and temporal resolution constraints in ensuring model robustness.

## 5. Conclusion

This comprehensive analysis of DMD in respiratory flow modeling revealed its dual strengths and limitations across three applications. The method achieved 85% Bhattacharyya correlation with ground-truth particle deposition patterns and showed strong agreement in fluid field reconstruction within trained parameter regimes, significantly reducing computational costs. DMD was particularly effective at extrapolating dominant flow features and preserving large-scale structures such as recirculation zones, and it demonstrated robustness in its reconstruction capabilities. As a comparison, the execution time for a full particle–fluid dynamics model with two-way coupling, utilizing 24 Dual Intel Xeon Gold 6226 CPUs @ 2.7 GHz, DDR4-2933 MHz DRAM, and 32 GB of RAM, required ~60 h to simulate a complete 2.4-second breathing cycle on a mesh with 60,204 cells and 177,802 mesh vertices. Conversely, a DMD-based interpolation of both particle trajectories and the fluid field, performed using 8 CPUs and 32 GB of RAM, achieved the same reconstruction in ~10 min. These findings position DMD as a promising reduced-order modeling framework for efficiently approximating respiratory flow and particle dynamics, supporting future applications where rapid, geometry-specific simulation is needed. However, its ability to interpolate intermediate inlet conditions, such as the untrained +10% inlet flow case, highlighted inherent constraints when applying DMD to complex geometries. The 20% parameter interval between training points led to localized errors exceeding 70% and a mean relative error across all time steps of ≈ 20%, underscoring that while DMD effectively projects nonlinear systems into linear Koopman spaces, geometrically induced flow complexities demand tighter parameter spacing for reliable interpolation. As a result, its applicability as an interpolator in sparsely sampled parametric simulations is limited.

Nonetheless, direct particle interpolation between the high-fidelity simulations at 100% and 120% inlet volume flow rates performed comparably to the direct DMD reconstruction of the full high-fidelity simulation, achieving a Bhattacharyya correlation coefficient of 80%.

While the proposed DMD and Koopman-based methods demonstrate promising reconstruction capabilities, several limitations remain. First, the simulations are limited to a single geometry and breathing condition, constraining generalizability. Second, particle–particle interactions were not modeled beyond two-way coupling with the fluid, potentially affecting accuracy in high-density aerosol environments. Third, the interpolation framework assumes linearity in the reduced-order space, which may not hold for all dynamic regimes. Future work will address these limitations through broader parametric variation, incorporation of subject-specific airway geometries, and development of non-linear reduced-order models and multi-resolution approaches.

These findings suggest that DMD’s current utility lies primarily in regime-specific reconstruction rather than broad parametric prediction in anatomically intricate domains. Future implementations could combine DMD with adaptive parameter sampling or physics-informed constraints to address these gaps, potentially enabling real-time analysis of patient-specific aerosol deposition without sacrificing critical flow dynamics. Incorporating such strategies may extend the applicability of reduced-order modeling frameworks like DMD in clinically relevant respiratory simulations, where computational efficiency and spatial resolution are both required to capture physiologically meaningful flow and deposition patterns.

## Figures and Tables

**Table 1:** Maximum and mean relative error across all snapshots.

Metric	Base	+20%	−20%	Interpolation
Maximum Error	13%	11%	13%	70%
Mean Relative Error	10%	11%	10%	20%
Outlets Max Error	2%	1.5%	2%	17%
Outlets Mean Error	1%	0.5%	1%	6%
Walls Mean Error	2.5%	1%	1%	9%

## Data Availability

Data will be made available on request.
